# A systematic review of economic analyses of telehealth services using real time video communication

**DOI:** 10.1186/1472-6963-10-233

**Published:** 2010-08-10

**Authors:** Victoria A Wade, Jonathan Karnon, Adam G Elshaug, Janet E Hiller

**Affiliations:** 1 Discipline of Public Health, The University of Adelaide, North Tce., Adelaide, 5005, Australia; 2 Hanson Institute, Institute of Medical and Veterinary Sciences (IMVS), Frome Rd., Adelaide, 5000, Australia

## Abstract

**Background:**

Telehealth is the delivery of health care at a distance, using information and communication technology. The major rationales for its introduction have been to decrease costs, improve efficiency and increase access in health care delivery. This systematic review assesses the economic value of one type of telehealth delivery - synchronous or real time video communication - rather than examining a heterogeneous range of delivery modes as has been the case with previous reviews in this area.

**Methods:**

A systematic search was undertaken for economic analyses of the clinical use of telehealth, ending in June 2009. Studies with patient outcome data and a non-telehealth comparator were included. Cost analyses, non-comparative studies and those where patient satisfaction was the only health outcome were excluded.

**Results:**

36 articles met the inclusion criteria. 22(61%) of the studies found telehealth to be less costly than the non-telehealth alternative, 11(31%) found greater costs and 3 (9%) gave the same or mixed results. 23 of the studies took the perspective of the health services, 12 were societal, and one was from the patient perspective. In three studies of telehealth to rural areas, the health services paid more for telehealth, but due to savings in patient travel, the societal perspective demonstrated cost savings. In regard to health outcomes, 12 (33%) of studies found improved health outcomes, 21 (58%) found outcomes were not significantly different, 2(6%) found that telehealth was less effective, and 1 (3%) found outcomes differed according to patient group. The organisational model of care was more important in determining the value of the service than the clinical discipline, the type of technology, or the date of the study.

**Conclusion:**

Delivery of health services by real time video communication was cost-effective for home care and access to on-call hospital specialists, showed mixed results for rural service delivery, and was not cost-effective for local delivery of services between hospitals and primary care.

## Background

Telehealth is the delivery of health care services at a distance, using information and communication technology (ICT). Telehealth became a separate field of study from the 1970's[1], and innovation increased from the 1990's, due to the development of new technologies such as cellular phones and the internet[2]. The field was originally known as telemedicine, but was later broadened to telehealth[3], although these terms continue to be used interchangeably. It is a subset of e-health, which encompasses all uses of ICT in health, including electronic records and decision support systems, however telehealth is particularly characterised by the geographical separation of patient and provider[4]. Tulu[5] categorises telehealth according to its purpose, such as clinical, educational or administrative; its healthcare discipline area; the environmental setting; the type of communication infrastructure used; and the delivery modality. Telehealth applications are very diverse, ranging from home care for chronic diseases, to remote primary care and subspecialist services such as paediatric cardiology. Telehealth can be delivered synchronously, also known as real-time, where the participants interact with each other simultaneously, and asynchronously, also known as store-and-forward, in which information such as X-rays or photographs are collected, transmitted, and then utilised at a later time.

Economic analysis is of central importance to telehealth because the main rationales for its introduction have been to decrease the cost of delivering health care, make more efficient use of the health workforce, and improve timely and equitable access to services. Expansive promises have been made about the potential to achieve these ends. For example Cusack[6]modelled cost savings of $4.3 billion a year if telehealth were implemented to facilitate consultations between healthcare providers in the USA, and this is without considering savings associated with the provision of care direct to the patient. However, the ability to deliver these results needs to be tested in the real world.

Three reviews published in 2000 covered the early economic analyses of telehealth[7-9], and each reported that the studies were methodologically flawed; lacking appropriate outcome measures, consistency and clarity. Thus, no conclusions could be made about the cost-effectiveness of telehealth at that time. In 2001, Roine[10] included economic assessments in a general review of telemedicine, and noted that most were poor quality cost comparisons of short term pilot projects, however there was evidence for cost savings in teleradiology. Whitten's review in 2002[11] appraised the quality of telehealth articles containing cost data, also finding that most were simple cost comparisons, with no definitive comparison of telemedicine to traditionally organised care. They also noted the difficulty of generalising results of individual economic studies due to the variability of applications and the effect of unique local factors on each telehealth service. Jennet reviewed the broader socio-economic impacts of telehealth in 2003[12] and combining cost, cost-effectiveness and health system utilisation measures into one category, concluded that there was evidence of benefit of at least fair to good quality in paediatrics, geriatrics, home care, radiology, mental health and rehabilitation. Hailey's review[13] of the benefits of telemedicine in 2004 rated 25 health economic analyses, using Drummond's criteria[14], finding 13 studies met 5 or more criteria and were rated as fair to good. These indicated cost savings in radiology, geriatric care, and intensive care, and conflicting evidence in dermatology.

From 2004, telehealth reviews diverge into specific areas of clinical practice, some of which contained economic outcomes. Two reviews of telepsychiatry concluded that cost-effectiveness could not be demonstrated because the volume of consulting was too low[15,16], while another found conflicting results of both increased and decreased costs[17]. Brebner[18] reviewed telehealth provision of accident and emergency support to primary care, and found seven studies with economic data, all indicating cost-effectiveness, however he concluded that the case was far from proven. A review of the use of telehealth in intensive care units found two clinical trials which showed cost savings[19]. Pare [20] summarised a number of reviews of home care for chronic disease, and reported that very few detailed economic analyses had been done, leading to no confirmation of economic viability. However, in regard to heart failure, Martinez concluded that home monitoring reduced costs of hospital admissions[21], and Seto reported initial costs, but substantial long term cost savings[22]. Recently, Bergmo[23] reviewed the quality of economic evaluations in telemedicine, and echoed the earlier findings of highly diverse evaluations, many of which did not adhere to standard economic evaluation techniques. Specifically, statistical, sensitivity, and marginal analyses, and information on the perspective of the studies were often lacking. Whereas this review pointed out methodological deficiencies, it did not aim to draw conclusions about the cost-effectiveness of telemedicine.

These reviews have covered a broad variety of telehealth applications, from synchronous videoconferencing, to remote monitoring, telephone follow-up, call centre advice lines, email and web-based systems. Each mode of telehealth has requirements for particular technology, staffing, services, and means of organisation, and consequently has different cost components, and can deliver particular types of outcomes. Not surprisingly, it has been difficult to compare results across studies and offer consistent guidance for cost-effective health services development. Therefore this review will focus on one mode of telehealth: real-time or synchronous video communication.

Real-time video can be regarded as the traditional form of telehealth, and despite new developments such as interactive software for chronic disease management, "smart houses" to measure resident's activity levels, and a range of monitoring devices such as scales, glucometers, and peak flow meters now able to send data remotely, real-time video remains in common use across a wide range of disciplines, particularly in mental health, primary care, specialist consulting, and multidisciplinary teamwork. It has particular requirements, and hence associated costs, for video screens, connectivity of sufficient bandwidth and reliability for real time communication, physical space at each location, and health providers' time to deliver the services. Research has been able to directly compare remote consultations with in-person consultations because the health providers' activities are similar to usual care. The intention of this review is to synthesise the economic analyses of this distinct form of telehealth, and determine whether conclusions can be made about its value.

## Methods

### Search Strategy

The following electronic databases were searched using these strategies:

• MedLine: ("Telemedicine"[Mesh] OR "Videoconferencing"[Mesh] OR "Telemetry"[Mesh] OR telehealth[Title/Abstract] OR telemedicine[Title/Abstract]) AND (cost[Title/Abstract] OR economic[Title/Abstract]) limited to English language and Humans.

• PsycInfo: (telehealth OR telemedicine OR videoconf* OR telemetry) AND (cost OR economic), limited to humans and peer reviewed journals

• CINAHL: (Telehealth OR telemedicine OR videoconf*) AND (cost OR economic)

• Scopus: (telemedicine OR telehealth OR videoconf*) AND (cost OR economic) for title, abstract or keywords, limited to articles

• Cost Effectiveness Analysis Registry (CEAR), produced by the Center for the Evaluation of Value and Risk in Health (CEVR), at the Institute for Clinical Research and Health Policy Studies at Tufts Medical Center: searched by the individual words telemedicine, telehealth and video

• NHS Economic Evaluations Database (NHS-EED): telehealth OR telemedicine OR videoconf*

The time frame for all searches was from the commencement of the databases until June 2009, or 2009 alone, when the database did not allow months to be specified.

In addition, review articles which were primarily about economic analysis of telehealth services, or included an economic component in the review had their reference lists searched for additional articles.

### Inclusion and Exclusion Criteria

Inclusion and exclusion criteria were developed to ensure the review consisted of economic analyses relevant to direct patient care.

#### Inclusion Criteria

• Economic evaluations of telehealth services which used synchronous video communication as the major mode of delivery

• Telehealth services which directly delivered patient care, either provider to patient or provider to provider, if the latter application was in the context of direct patient care.

• Health economic analyses that included or cited health outcomes data obtained from telehealth services. This data included patient health status and health utilisation measures, but not patient satisfaction measures. Analyses were accepted as cost minimisation if they either produced original data or cited evidence that the outcomes of health care delivery were equivalent or non-inferior for synchronous video compared to usual care. Citations were checked to see that they were of methodologically sound research, and could be validly generalised to the particular telehealth setting, to determine whether a cost-minimisation analysis could be undertaken.

#### Exclusion Criteria

• Articles that did not contain economic data ie commentary, theory, study design and methodology articles.

• Articles about telehealth services that were not about direct patient care ie education, administration and social uses of the telehealth infrastructure. This included articles about health provider reimbursement and business models for telehealth services.

• Articles about telehealth services with no or very little real time video component ie telemonitoring, store-and-forward of images, telephone, SMS, or email applications.

• Cost analyses with no health outcomes data.

• Economic analyses where the only health outcomes were satisfaction data, or where the health outcomes were not collected equally across comparison groups.

### Quality Rating

Articles in the review were assessed according to Chiou's grading system of the quality of health economic analyses[24]. This system contains 16 weighted items developed by an expert committee; some items are applicable to research in general, such as the clarity of the study objective, use of reliable and valid health outcome measures, the methodology for data abstraction, use of statistical analysis, discussion of bias, justified conclusions and disclosure of funding source. Other items apply particularly to economic analysis, such as a statement and justification of the economic perspective, the use of sensitivity or incremental analysis, the length of the time horizon, the use of discounting, the methodology for cost estimates and measurements, and the choice of economic model. The reliability and validity of this method was previously established by testing the scale with a sample of 60 health economists who each rated the same set of economic analyses using the criteria. By contrast, other methods for assessing the quality of economic analyses, such as Drummond's criteria[14], or the checklists used by the Mair[8] and Bergmo[23] reviews have been developed by individual experts. VW and JK graded 3 studies using Chiou's criteria in order to reach consensus on the interpretation of the criteria, and VW graded the remaining papers.

## Results

### Results of Search

Using the strategies described above, MedLine produced 1187 search results, PsycInfo 286, CINAHL 393, Scopus, 1887, CEAR 10, and NHS-EED 197.

**Figure 1:**
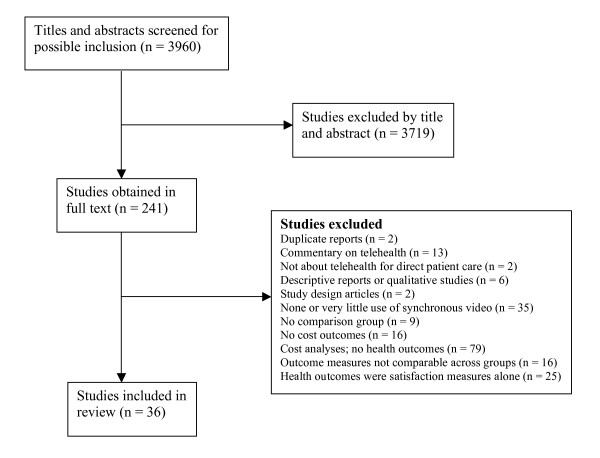
**Flow chart of study inclusions**.

The reference lists of 24 review articles that were entirely or in part about the economic analysis of telehealth were searched, and did not produce any additional studies that met the criteria. A flow chart of exclusions is shown in Figure 1

### Study Characteristics

Table 1 summarises the characteristics of the 36 studies included in the review. The clinical disciplines delivered by the telehealth services were diverse: dermatology (7), mental health (6), paediatric cardiology (4), home nursing (4), intensive care (2), emergency medicine (2), and neurology (2), and single studies in six medical disciplines (infections diseases, internal medicine, general practice, cardiology, oncology, and pain management), and four surgical disciplines (ear nose and throat surgery, orthopaedics, gynaecology, and neurosurgery).

**Table 1:** Summary of Economic Analyses

Article	Service Setting	Clinical Discipline	Study Type	Ec Analysis Type & Quality Score	Technology & Connectivity	Perspective	Cost Outcomes	Telehealth outcome vs usual care
Ruskin 2004[51]	Rural outpatient	Psychiatry	RCT	CMA 84	Computer, ISDN	Health service	More	Same

Modai 2006[72]	Rural outpatient	Psychiatry	ProspCC	CCA 45	Not specified, ISDN	Health service	More	Same

O'Reilly 2007[73]	Rural outpatient	Psychiatry	RCT	CMA 54	VC, ISDN	Health service	Less	Same

Shore 2007[29]	Rural outpatient	Psychiatry	DiagAcc	CMA 57	VC, ISDN	Health service	Mixed^1^	Same

Crow 2009[35]	Rural outpatient	Psychology	RCT	CEA 77	Not specified, T1 line	Societal	Less (HS less)	Less^2^

Persaud 2005[53]	Rural outpatient	Psychiatry & dermatology	RetCoh	CMA 46	Not specified, 384 kbits/sec	Societal	Less (HS more)	Same

Oakley 2000[74]	Rural outpatient	Dermatology	RCT	CCA 15	Computer, ISDN	Patient	Less	Same

Loane 2001a[32]	Rural outpatient	Dermatology	RCT	CMA 70	Computer, ISDN	Societal	Less (HS more)	Same

Loane 2001b[59]	Rural outpatient	Dermatology	RCT	CMA 40	VC, ISDN	Health service	More	Same

Chua 2001[36]	Rural outpatient	Neurology	RCT	CCA 25	VC, ISDN	Health service	More	Less

Bergmo 1997[52]	Rural outpatient	Ear nose & throat	RetCoh	CMA 68	VC, ISDN	Societal	Less (HS less)	Same

Ohinmaa 2002[33]	Rural outpatient	Orthopaedics	RCT	CMA 71	VC, ISDN	Societal	Less (HS more)	Same

Bishai 2003[62]	Rural outpatient	Gynaecology	DiagAcc	CCA 84	VC, T1 line	Societal	More	More

Pronovost 2009[31]	Rural outpatient	Pain management	RCT	CMA 93	VC, broadband	Societal	Less (HS more)	Same

Johnston 2000[39]	Home care	Nursing	RCT	CCA 19	Not specified Not specified	Health service	Less	Same

Smith 2002[42]	Home care	Nursing(CPAP)	DiagAcc	CMA 25	Custom, PSTN	Health service	Less	Same

Bohnenkamp 2004[41]	Home care	Nursing	NRT	CCA 35	TV, PSTN	Health service	Same	More

Finkelstein 2006[38]	Home care	Nursing	RCT	CCA 67	TV, PSTN	Health service	Less	More

Chan 2000[58]	Residential care	Dermatology	DiagAcc	CCA 32	VC, ISDN	Health service	Less	Less^3^

DeMaio 2001[43]	Home care	Infectious diseases	B&A	CMA 15	Vphone, ISDN	Health service	Less	Same

Jerant 2001[37]	Home care	Cardiology	RCT	CCA 67	Not specified PSTN	Health service	Less	More

Eron 2006[40]	Home care	Internal medicine	ProspCC	CCA 67	Not specified PSTN	Health service	Less	More

Rendina 1997[75]	Rural inpatient	Paediatric Cardiol	B&A	CBA 56	VC, T1 line	Health service	Less	Same

Sable 1999[55]	Rural inpatient	Paediatric Cardiol	DiagAcc	CBA 27	Computer ISDN	Health service	Less	More

Sicotte 2004[56]	Rural inpatient	Paediatric Cardiol	DiagAcc	CEA 63	VC, ISDN	Health service	More	More

Dowie 2007[71]	Rural inpatient	Paediatric Cardiol	ProspCoh	CCA 85	VC, ISDN	Societal	Mixed^4 ^(HS more)	More

Ehlers 2008[76]	Rural inpatient	Neurology	EcModel	CUA 65	Computer, Not specified	Societal	Less (HS more)	More

Wong 2006[64]	Rural inpatient	Neurosurgery	RCT	CCA 64	VC, ISDN	Health service	More	Same

Noble 2005[61]	Rural inpatient	Emerg med	RCT	CCA 60	Not spec, ISDN	Societal	More	Same

Duchesne 2008[57]	Rural inpatient	Emerg med	B&A	CCA 34	VC, T1 line	Health service	Less	More

Kunkler 2007[60]	Rural inpatient	Oncology	RCT	CMA 64	VC, ISDN	Health service	Less	Same

Loane 2000[45]	Hosp to PCare	Dermatology	RCT	CMA 54	VC, ISDN	Health service	More	Same

Wootton 2000[46]	Hosp to PCare	Dermatology	RCT	CCA 81	VC, ISDN	Societal	More	Same

Jacklin 2003[47]	Hosp to PCare	General practice	RCT	CCA 92	Computer, ISDN	Societal	More	Same

Rosenfeld 2000[50]	Specialist on-call	Intensive Care	B&A	CCA 80	Computer, Not specified	Health service	Less	More

Breslow 2004[49]	Specialist on-call	Intensive Care	B&A	CCA 53	VC, T1 line	Health service	Less	More

B&A = before and after study, DiagAcc = diagnostic accuracy study, EcModel = economic modelling, NRT = non-random trial, ProspCC = prospective case control, ProspCoh = prospective cohort, RCT = randomised controlled trial, RetCoh = retrospective cohort study

ISDN = Integrated Services Digital Network, VC = videoconferencing, Vphone = videophone, Hosp to PCare = Hospital outpatients clinic to local health centre

CMA cost-miminisation analysis, CCA cost-consequences analysis, CBA cost-benefit analysis, CEA cost-effectiveness analysis

1. Cost more in 2003, and less in 2005

2. Less effective, but increased patient access to services

3. Worse diagnostic accuracy, but increased patient access to services

4. Cost less for neonates, more for other patient groups

Eighteen of the studies were randomised controlled trials, 6 were studies of diagnostic accuracy, 5 were before and after studies, 2 were prospective case control, 2 were retrospective cohort studies, and there was one each for the categories of non-random trial, prospective cohort and economic modelling.

### Health Outcome Measures

When the purpose of the telehealth service was primarily diagnosis and assessment of patients, diagnostic accuracy was used as an outcome, as well as the related measure of the percentage of patients referred for further investigation. However, when patient management was the main intention, a variety of validated instruments were used to assess patient status, including the SF-36, the Global Assessment of Functioning, or the Brief Psychiatric Rating Scale. In some studies measures were chosen for particular settings, such as time taken to achieve self-care, patient adherence to treatment, or quality ratings of medical decisions. In heart failure and intensive care settings, mortality was also used as an outcome. Various healthcare usage rates were reported, such as hospital length of stay, re-admissions, and attendance at emergency departments and outpatient clinics. In tele-dermatology, a measure often used was the percentage of patients requiring further consultations: a proxy for patient outcome, as the dermatological problem is taken as resolved if the patient no longer required specialist care.

### Economic Analysis Characteristics

The economic analyses were classified by their health outcome measures, with 18 studies assessed as being cost-consequences, reflecting the diversity of measures described above, 13 cost-minimisation, two cost-effectiveness, two cost-benefit and one cost-utility study. Six of the cost-minimisation studies relied on citations of previous work for health outcomes data; in five of these the authors cited their own work in the same clinical settings[25-29], and one study cited a very similar setting[30], therefore these citations were taken as valid evidence of equivalence. 23 studies took the perspective of the health services, 12 were societal, and one was from the patient perspective alone. Using Chiou's criteria for quality assessment, it was apparent that Criterion 4, which states that if the analysis is carried out on a subgroup then the subgroups should be pre-specified at the beginning of the study, was not applicable in 35 of the 36 cases. This criterion was therefore deleted from the grading, leaving the total quality scores out of 99 rather than 100.

Quality varied widely ranging from 15 to 93 points, with four studies rated at 25 or less. The criterion least often fulfilled was having an analytic horizon that allowed time for all relevant and important outcomes to be assessed. This was achieved by only 7 (19%) of studies. Other lower scoring criteria were including a justification for the perspective of the study (36%), and performing an incremental analysis between alternatives for resources and costs (33%). Most studies met the criteria of presenting their objectives in a clear, specific and measurable manner (90%) and provided conclusions that were justified and based on the study results (78%). There were no clear associations between the quality of the studies and the clinical discipline, the organisational model of care, or the year of publication.

### Costs and Effects

Overall, 22 (61%) of the studies found telehealth to be less costly than the non-telehealth alternative, 11 (31%) found greater costs, 2 (6%) gave mixed results and in one (3%) the costs were the same. The 8 studies that reported lower costs from the societal perspective were looked at in more detail, and 6 of these showed higher health services costs. Excluding the single study that reported only on the patient perspective, the results from the health service perspective were 17 (49%) found telehealth more costly, 16 (46%) less costly, 1 (3%) found costs changed from more to less over time, and 1 (3%) found costs to be the same. These differences are largely accounted for because in delivering services to rural areas, the health services paid more for telehealth, but the societal perspective demonstrated cost savings due to reductions in patient travel[28,31-34]. In studies where both the societal and the health services perspective showed lower costs, the health services were paying for health care workers to travel[30,35].

Regarding health outcomes, 12 (33%) of studies found improved health outcomes, 21 (58%) found outcomes were not significantly different, 1 (3%) found outcomes differed according to the patient group, and 2 (6%) found that telehealth was less effective. Both of the latter studies were of outpatient services to rural areas; one found that more investigations were ordered in a telehealth neurology clinic[36], and the other delivered treatment of bulimia nervosa by videoconferencing, which was clinically effective, but less so than in-person treatment[35]. Neither of these services compromised patient safety. Table 1 sets out the cost and effectiveness results of all studies.

The 18 RCTs showed evenly divided results, with 9 studies reporting telehealth to be more costly and 9 less costly than the non-telehealth comparison, and 15 reported the health outcomes to be the same. The 18 non-RCTs showed results more favourable to telehealth, with 12 reporting telehealth to be less costly, 3 more costly, and 3 giving equal or mixed results. Ten reported improved and 8 similar health outcomes.

There was no indication that the more recent studies were more likely to be cost saving, or to offer improved outcomes, nor were there patterns by clinical discipline. However, when the studies were grouped into five different organisational settings, patterns of costs and effects became apparent:

#### 1. Home care

There were 8 economic analyses of telehealth in this group, 7 delivered to the home, and one to an aged care facility, which was the patients' normal place of residence. All were conducted from the perspective of the health care services, with 7 showing cost savings and one no difference in costs. Three studies were RCTs, and these reported reduced hospital admissions[37], reduced transfer to nursing home care[38], and greater satisfaction, but no other differences in outcomes[39]. Two of the non-random comparisons found the telehealth groups improved their functioning more rapidly[40,41]. The only study on delivery of services to an aged care facility showed a mixed result of increasing accessibility to dermatology services, but reduced diagnostic accuracy. Three of the studies in this group had low quality ratings, of 25 or less, for their economic analysis; two were small scale feasibility projects[42,43]), and the third included an economic analysis as part of a larger study[44].

#### 2. Specialist consultation to primary care

These studies of telehealth from the hospital to local primary care services were all RCTs, two for dermatology services and one for a variety of specialist consultations. In each study, the telehealth intervention involved the patient sitting with a general practitioner whilst consulting with the specialist at a hospital via video link, and the comparison was usual care in which the patient was referred to the hospital for an outpatient consultation. All studies showed similar patient outcomes and increased costs for the health services[45-47]. Two of the economic analysis reported modest time and money savings for patients, but from the societal perspective, telehealth remained more costly. Although two of these studies[45,48] reported that they used both urban and rural settings, the distances concerned were of the order of 10 to 20 kms, so they have been classified as local care.

#### 3. Specialist on-call to hospital

Both of these before and after studies were conducted in intensive care units, where telehealth provided off-site intensivist coverage to hospital wards that had not previously had this service available. Each found lower costs and reductions in patient mortality when the telehealth service was operating; additionally Breslow[49] reported shorter hospital stays, and Rosenfeld reported lower complications[50].

#### 4. Rural outpatient care

This was the largest group, with 14 studies, including all six of the services delivering mental health care, 4 dermatology services, ENT, orthopaedics, gynaecology, and pain management. Nine of the studies were RCTs, and of these, 5 showed telehealth was less costly, and 4 showed increased costs. In regard to patient outcomes, 6 RCTs reported that telehealth was as effective as usual care, and 2 reported reduced effectiveness. Of the 5 non-randomised studies, one showed reduced costs and 4 showed increased costs, and for patient outcomes, one showed better outcomes and 4 found equal outcomes. When sensitivity analyses were done, the cost outcomes depended on the distance between sites, or the frequency of use that was made of the telehealth facilities. Ruskin[51] found that telepsychiatry was more expensive if the psychiatrist needed to travel less than 22 miles to an outlying clinic. Bergmo[52] calculated that ENT teleconsultation became cost-effective if more than 52 patients were seen in a year, Ohinmaa[33] conducted a similar analysis showing that 80 patients a year were needed to break even for orthopaedic outpatient teleconsultations, and Persaud[53] calculated that at present workloads, the telehealth option was more expensive, but would become less expensive at a practically attainable level. One of the studies in this group was a limited analysis from the patient perspective only, and it had a low score (of 15) on the economic analysis quality rating[54].

#### 5. Rural inpatient care

These 9 studies involved specialty clinicians in a central tertiary level hospital consulting with health providers in rural and regional hospitals about patients who were either admitted or were emergency patients, known as the hub-and-spoke model. Four of the studies were in the clinical area of paediatric cardiology. Three studies were RCTs and all showed similar health outcomes to the non-telehealth alternative, with 2 demonstrating increased costs and one lower costs than usual care. The 6 non-random comparison studies found results that were more favourable to telehealth, with 5 reporting better and one equal health outcomes, and 5 reporting reduced costs, and one increased costs. The improved health outcomes included reduced numbers of patients transported out of rural areas[55,56], and reduced time to transportation[57].

### Technology

Videoconferencing equipment was used most commonly, in 18 studies, followed by computers (7), home televisions (2), videophones (1), a custom-designed unit (1), and unspecified equipment (7). Sicotte[56] noted that equipment was 78% of the total cost of the telehealth modality of delivery and Chan[58] stated that their viewstation was the single largest cost of their service. Persaud[53] and Loane[59] both reported that capital costs were the main reason that telehealth was more costly per consultation, although Loane[59] noted that equipment prices had reduced by the time of publication to the point that if updated costs were used in their analysis, telehealth would have been less costly than conventional care. Kunkler[60] reported that halving the technology cost would reduce their breakeven point from 40 to 20 multidisciplinary meetings per year, although Noble[61] and Ohinmaa[33] found that reducing the annual equipment cost had no impact on the overall cost outcomes.

For connectivity, 22 studies used Integrated Services Digital Network (ISDN) lines, which deliver 128 kilobits/second of data each, and four studies used T1 lines, which are considerably more costly, and can carry 1.5 megabits/second of data. Five studies, all of which were home care, used the PSTN (Public Switched Telephone Network), which is the standard low-bandwidth telephone line supplied to households, and three studies did not specify the connectivity. One study reported using "broadband"[31], and it was assumed that this meant Digital Services Lines (DSL). Connectivity was a significant cost for many telehealth services, with Bishai[62] reporting T1 line charges as their largest single cost, and Chua[36] noting that the hourly cost of ISDN communication was almost identical to the hourly pay of a consultant neurologist. Meilonen[63] commented that differences in telecommunications costs made telehealth cost-saving in some countries and uneconomical in others, and Shore[29] found that telehealth changed from being more costly in 2003 to less costly in 2005, due to reductions in ISDN line charges. However, sensitivity analyses in some studies showed the cost of connectivity to be irrelevant, for example Jacklin found that telehealth remained more expensive even if the cost of telecommunications was reduced to zero, but on the other hand, Crow[35] found that despite using T1 lines, telehealth was always less expensive than usual care.

Three studies reported significant technical problems with their video communication. Persuad[53] said that 29% of their teleconsultations had technical problems, which took an average of 7.2 minutes each to fix, Sable[55] found 8 of the 60 transmitted real time echocardiograms had significant technical problems and 5 could not be transmitted at all, and Wong[64] noted an unacceptably high failure rate of 30.1% for videoconferencing.

## Discussion

The literature on telehealth is extensive, however there are a relatively small number of economic analyses that met the review criteria. Identification and review of evidence of equivalence for all economic studies that did not claim or cite equivalence was beyond the capacity of this study, and so a pragmatic approach was taken that excluded economic studies that either did not claim equivalence or provided no direct or indirect (cited) evidence of effectiveness. Cited evidence of equal health outcomes was reviewed to establish that the evidence was relevant to the setting of the economic analysis. This approach resulted in the exclusion of some well-conducted cost analyses[65-68]. Identification of evidence around the effect of the services analysed in these studies would increase the pool of evidence on the cost-effectiveness of telehealth services.

Those studies in which the only health outcomes reported were patient satisfaction ratings were also excluded because telehealth studies report almost uniformly high patient satisfaction, which is likely to be a positive bias due to social desirability and acquiescence[69]. Finally, studies were excluded in which the health outcomes were not comparable, such as where participants receiving telehealth were asked to rate the success or accuracy of the teleconsultation, but this was not done for the usual care comparator.

Quality assessment using Chiou's criteria[24] gave similar results to those of Bergmo's recent review of economic analyses in telehealth[23]. Bergmo reported that studies were most lacking in providing information on the perspective of the study, statistical comparison, sensitivity analyses, and marginal analysis. The criterion least fulfilled in this review, allowing sufficient time to assess all important outcomes, was not used by Bergmo, who developed a new set of criteria based on issues mentioned in the telehealth literature. By comparison, Chiou's criteria were developed and validated by a study of health economists. Also in line with earlier reviews[11,23], there was great variability in methodology and outcome measures. The majority of studies were cost-consequences analyses, utilising outcomes specific to each service, and therefore a quantitative meta-analysis could not be attempted.

Overall, nearly two thirds of the studies showed cost savings in utilising telehealth, according to the perspectives adopted by each. In particular, whenever the patient perspective was assessed, telehealth was found to be cost-saving, however when the health services perspective was considered alone, which was possible for 35 of the studies, the proportion reporting cost savings reduced to half. Most studies showed similar health outcomes, about a third showed improved outcomes, and only two indicated that telehealth was less effective. Surprisingly, there was no obvious trend for the more recent studies to indicate more cost savings, which was expected due to the reduction in technology costs over time. When seeking to ascertain the particular circumstances within which telehealth was most cost-effective, the organisational model of care was found to be the most relevant factor.

### Organisational Models

The studies that delivered home care via real time video produced cost savings; an interesting finding because reviews of tele-homecare have mainly used telephone and/or telemonitoring[20,22,70]. The videoconferencing units used between health care services have been too expensive for home installation, but this group of studies indicates that various options for home video communication are effective and efficient, allowing for a greater range of telehealth services to be delivered to the home than is possible with audio or data communication alone. If the two short-term feasibility studies are taken out of consideration, one is still left with six studies that show similar results.

Secondly, bringing the expertise of on-call intensive care specialists to hospital wards via telehealth was found to lower costs and improve patient outcomes. This model of care was not a substitution of one form of delivery for another, but an addition to the expertise available to an existing service. However, there were only two studies in this group, and both were in intensive care, so further work is needed before conclusions can be generalised to other areas of hospital care.

When video communication was used between hospitals and local primary care services, there were increased costs due to medical staff time, and additional costs for equipment and connectivity. In each study the patients saved a modest amount of money on travel and reduced time off work, but this was not sufficient to offset the greater costs to the health services. The patient outcomes were similar to usual care, therefore it appears that this model of health service delivery is not economically viable.

Where health care is delivered to rural and remote areas the economic outcomes are variable for both inpatient and outpatient delivery of care. However in these settings cost may not be the only factor that determines implementation, because telehealth is also utilised to improve accessibility or timeliness of service delivery. Policies about equity of access, or local political factors, may contribute to a service that costs more, or is not as effective, being delivered to a rural area. For example, although treating bulimia by videoconferencing was less effective than face to face care, [35], in the real world there is very limited availability of this service in rural areas, so the options are no treatment or treatment that is still genuinely clinically useful. It was notable that four of the telehealth services to rural inpatients were for paediatric cardiology. This discipline may be particularly suited to telehealth because it has a combination of a small number of highly specialised clinicians, only available in tertiary referral hospitals, high costs of transporting infants and children for investigation and consultation, and families that would prefer treatment closer to home.

### Technology

The use of technology is one of the defining characteristics of telehealth, and the costs of technology were critical to the outcomes of many of the studies. The stand-alone videoconferencing equipment used in most studies could be very costly, and although prices have come down over the past decade, expensive tele-presence units with multiple screens and peripheral devices are now being marketed as new generation products. The equipment used in home care was much less expensive, utilising either the patient's own television or smaller video units.

Six of the seven home care studies used the PSTN, ie the patient's own telephone lines, which are a minimal cost to the health service because the patient has already paid for installation and line rental. The PSTN can be used for video communication, however the bandwidth is low, which results in a jerky, reduced quality image, due to low frame rates, however despite the quality of the image, these services were still able to produce improved patient outcomes.

The studies in this review do not reflect the recent advances in connectivity produced by widespread rollout of fast broadband or Digital Services Lines (DSL) with only one recent study utilising DSL[31]. This may be because of quality and reliability issues with DSL. Although both types of lines are digital, ISDN lines provide exclusive bandwidth to the purchaser, whereas with DSL the bandwidth is shared by all the users of the Internet Service Provider (ISP) that is supplying the connectivity. For DSL to provide good quality real time video, it needs to have reliable, high upload and download speeds, but if the ISP has over-sold their available bandwidth, and/or the health care service has not purchased enough bandwidth, DSL users will have the experience of poor quality and delays during busy times. New forms of connectivity, such as wireless broadband or fast mobile data services have the potential to make video-based telehealth more flexible and easier to install in a range of settings, including home care.

### Facility space

When using synchronous video communication, the patient and provider are, by definition, in two different places at the same time, therefore the cost of each location needs to be taken into account. When one of the locations is a home, for example when a specialist is on-call or working from home, or the service is being delivered to a patient's home, there is no additional cost for office space, but where both locations are health services, each location should be costed. For rural locations, it is often assumed that the patient can attend a local health service to have a telehealth consultation with no additional cost to the remote health service, but this assumes the service has spare rooms, booking, and reception facilities, which may not be the case.

### Health Workforce

One of the reasons that telehealth from a hospital to local primary health centre was more expensive than usual care was that a general practitioner was with the patient during the specialist consultation, hence more medical workforce time was used per visit[46,47]. Again, the precise model of care is important, because in serving remote areas this same approach may save costs by reductions in travel time, or improve patient outcomes through more immediate treatment[32,52]. There can also be savings in professional time if transfers are avoided, because health care workers are then not needed to accompany the patient. On the other hand if urban providers are expected to add telehealth onto their current workload for no extra recompense, some resistance would be expected. By contrast, in home care, the patient is usually unaccompanied and therefore only one health professional is consulting with them at a time. However, tele-home care can lead to additional use of health workforce if the telehealth visits are added on to, rather than substituting for, existing services[41].

However, when two health care providers see the patient at the same time, there is the potential for a knowledge transfer effect, leading to reduced specialist referrals, increased and/or improved patient management at the primary care level, and subsequent time and cost savings. This has been reported for primary care management of ENT problems[52], colposcopy [62], and skin conditions[59]. A learning effect leading to 5% less referrals to orthopaedic outpatient clinics was noted by one study [33], however this was estimated by local physicians rather than measured directly. Jacklin[47] hypothesised that joint GP-specialist consultations would lead to downstream savings through improved patient management, but their results did not support this, although the short follow up period of six months and the broad range of specialist disciplines involved may not have allowed enough time for a learning effect to be measurable.

### Waiting time

Telehealth can reduce the waiting time to receiving specialist care. For example it has been noted[71] that a visiting paediatric cardiology service was less than monthly, whereas a teleconsultation could be arranged within two days, and that telehealth reduced the time for an ENT consultation from four months to four to six weeks[52]. Researchers have suggested that earlier diagnoses have value in their own right, by providing reassurance, as well as from the health effects of earlier treatment[56]. In addition, long waiting times may lead to additional costs for interim care, or loss of productivity due to untreated conditions[46], however these issues were not assessed in the studies included in this review.

### Limitations

There were limitations in the quality of many of the economic analyses that met the criteria for this review, which in turn limits the strength of the conclusions. This is of particular importance because reducing cost and improving efficiency are key arguments for the introduction of telehealth. The validity of these analyses could be improved by:

• Longer term studies to detect potential longer term effects, such as decrease in health services utilisation that may occur if patient outcomes are improved.

• Measuring additional potential outcomes, such as knowledge transfer in provider to provider telehealth.

• Greater homogeneity in study methods and outcome measures, for example, increased use of QALYs.

• Standardising the method for assessing the quality of the economic analyses.

It was also not possible to perform a quantitative meta-analysis of the effects of telehealth because of the heterogeneity of the outcome measures. A higher proportion of the non-random comparisons showed lower costs and better outcomes than did the RCTs, suggesting that the studies with a lower level of evidence are biased in favour of telehealth.

Generalisatiblity is a problem for telehealth research as a whole, due to variability in clinical disciplines, environmental settings, workforce and health care financing. This review attempts to deal with this by considering only real time video communication, which has similar infrastructure and ways of clinical practice, and also by grouping the results into similar organisational settings; however this variability means that generalisation should still be considered with caution. Although there are patterns in the results by organisational setting, numbers in these groups are low, and the conclusions would be strengthened by additional research in each area.

Finally, concluding the search at mid-2009, together with the delay between research and publication, has meant that more recent research, which could use higher bandwidth and less costly connectivity, was not yet available for inclusion.

## Conclusion

Reviewing 36 economic analyses of the delivery of health services by synchronous video communication indicates that this form of telehealth can offer value to health care, and it suggests that key factors associated with this are the settings and particular models of health service delivery. The health outcomes of the patients were either equal to or better than conventional care, with two minor exceptions that did not compromise quality of care. Therefore the decision as to whether or not to introduce a telehealth service can be made using cost-effectiveness criteria and consideration of the model of care.

It is concluded that synchronous video delivery is cost-effective for home care, and for on-call hospital specialists, and it can be cost-effective for regional and rural health care, depending upon the particular circumstances of the service. However, it is not cost-effective, from the health services perspective, for local delivery of service between hospital specialists and primary care, particularly due to additional health care staffing. Across settings, equipment and connectivity costs have been major factors in setting up telehealth services, but even as these costs reduce, this will not necessarily make telehealth more cost-effective, unless the other factors such as health workforce and facility space are also addressed.

Improvement in the quality of economic analyses is also needed to provide data for more accurate modelling of the effects of widespread introduction of telehealth into the health care system.

## Competing interests

VW declares that she has a competing interest, being the Medical Director of Design Networks Pty Ltd, a company that supplies telehealth services. This position is not salaried, however VW has received reimbursement of expenses for conference attendance from the company. The company did not initiate or fund this work. The other authors declare that they have no competing interests.

## Authors' contributions

VW conducted the searches, analysed the data and wrote the article, JK analysed some of the data, supervised data analysis, and reviewed the article, AE supervised and reviewed the article, and JH supervised and reviewed the article. All authors read and approved the final manuscript.

## Pre-publication history

The pre-publication history for this paper can be accessed here:

http://www.biomedcentral.com/1472-6963/10/233/prepub
